# Bilateral ovarian cystic teratomas with a left Sertoli Leydig cell tumor: A case report

**DOI:** 10.1097/MD.0000000000042462

**Published:** 2025-05-09

**Authors:** Yingtong Zhang, Shimei Li, Jie Luo, Ping Sun, Yulan Song

**Affiliations:** a Department of Endocrinology, The First Affiliated Hospital of Guangdong Pharmaceutical University, Guangzhou, Guangdong, China; b Department of Gynaecology, The First Affiliated Hospital of Guangdong Pharmaceutical University, Guangzhou, Guangdong, China; c Department of Pathology, The First Affiliated Hospital of Guangdong Pharmaceutical University, Guangzhou, Guangdong, China.

**Keywords:** amenorrhea, hyperandrogenism, ovarian teratoma, ovary, Sertoli Leydig cell tumor

## Abstract

**Rationale:**

Ovarian mature cystic teratoma (MCT) is the most common ovarian germ cell tumor, and it is typically unilateral, although bilateral MCTs have been observed. Sertoli Leydig cell tumors (SLCTs) are rare ovarian tumors that can cause hyperandrogenemia. When patients have these 2 tumors at the same time, it is highly challenging to diagnose them.

**Patient concern:**

A 32-year-old female patient who complained of a pelvic mass for 1 year and amenorrhea for 6 months with high testosterone and 17-hydroxyprogesterone levels was suspected of having a malignant ovarian tumor. Transvaginal ultrasound revealed mixed lesions in the bilateral adnexal areas, and the nature of the left slightly hyperechoic mass was not determined.

**Diagnoses:**

On the basis of the patient’s laboratory examination, imaging examination and pathological examination, she was diagnosed with hyperandrogenemia and bilateral MCTs with a left SLCT.

**Interventions:**

The patient underwent laparoscopic surgery to remove bilateral ovarian neoplasms.

**Outcomes:**

Testosterone levels decreased to 1.51 nmol/L on the 1st day after fertility-sparing bilateral ovarian tumor resection and to 0.73 nmol/L on the 5th day.

**Lessons:**

In conclusion, we reported a case of bilateral MCTs with left SLCT. The patient examinations supported the diagnosis of this rare disease. Once ovarian SLCT is diagnosed, the tumor should be surgically removed immediately, and the subsequent treatment plan should be selected according to the immunohistochemical results.

## 1. Introduction

Ovarian mature cystic teratoma (MCT) is a common benign reproductive system neoplasm composed of tissues from the ectoderm, mesoderm, and endoderm. MCT accounts for approximately 11% of ovarian neoplasms and 69% of germ cell tumors in young women.^[1]^ Most MCTs of the ovary are unilateral, with approximately 10% of cases occurring bilaterally.^[2]^ Sertoli Leydig cell tumors (SLCTs) are rare reproductive system tumors that produce hormones, account for <0.5% of ovarian neoplasms, and is mostly detected in young women under 30 years of age.^[3]^ Clinical manifestations such as irregular menstruation and virilization occur in 50% of cases, and estrogen manifestations occasionally occur in patients.^[4]^ In this case report, we present a case of bilateral MCTs and left SLCT in a 32-year-old female with elevated testosterone levels.

## 2. Case presentation

A 32-year-old Chinese woman, gravida 0, para 0, was admitted to the hospital with a chief complaint of the existence of a pelvic mass for over 1 year and amenorrhea for 6 months. The patient experienced menarche at the age of 12 years with irregular menstrual cycles. No familial history of malignancy was reported. Her abdominal ultrasound revealed a mass in the left adnexa, which was speculated to be an ovarian teratoma 1 year prior. Six months prior, the patient developed amenorrhea, accompanied by voice hoarseness, facial acne, and hirsutism on her vulva and 4 limbs. Therefore, the patient came to our hospital on December 13, 2023 and underwent a physical examination. According to the physical examination, she had hirsutism and clitoromegaly; a neoplasm approximately 2 × 2 mm in size could be seen at the cervical opening, and a palpable mass of 4 × 3 × 3 cm in the pelvic cavity was observed. The laboratory data revealed the levels of different factors, including hemoglobin 152 g/L (normal range 115–150 g/L), testosterone 25.05 nmol/L (normal range 0.38–1.97 nmol/L), and 17-hydroxyprogesterone (17-OHP) 6.45 ng/ml (normal range Follicular phase: ≤1.05; Ovulatory phase: 0.13–1.46; Luteal phase: 0.27–2.41; Table 1). Transvaginal ultrasound revealed mixed masses (M1) with similar echoes in the bilateral adnexal area. The sizes were 28 × 18 × 19 mm (right) and 40 × 29 × 30 mm (left), and a small liquid area could be observed locally. Another slightly hyperechoic mass (M2) with a size of approximately 43 × 31 × 36 mm was observed in the left adnexal area. Color Doppler flow imaging indicated no obvious blood flow signal in the mixed masses of the former (M1), which could be ovarian teratomas. The latter mass (M2) in the left adnexal area presented a low resistance blood flow signal. The nature of the tumor has yet to be determined. Contrast-enhanced magnetic resonance revealed that the size and shape of the bilateral adrenal glands were normal, and no abnormal enhancement foci were found. There were abnormal signal masses in the bilateral adnexal areas. It was considered to be of ovarian origin.

**Table 1:** Laboratory data before surgery and 1 day after surgery.

	Before	After	Reference values
Hemoglobin (g/L)	152	127	115–150
Testosterone (nmol/L)	25.05	1.51	0.38–1.97
Luteinizing hormone (mIU/mL)	7.17	7.99	Follicular phase: 1.80–11.78 Ovulatory phase: 7.59–89.08 Luteal phase: 0.56–14.00
Estradiol (pmol/L)	281.74	273.04	Follicular phase: 77.1–921.2 Ovulatory phase: 139.6–2381.8 Luteal phase: 77.1–1145.0
Progesterone (nmol/L)	2.90	＜0.32	Follicular phase: ＜0.318–0.954 Luteal phase: 3.82–50.56
AMH (ng/mL)	3.45	N/A	0.5–8.6
HE4 (pmol/L)	51.3	N/A	0–70.0
CA125 (U/mL)	4.6	N/A	0–35
CA199 (U/mL)	4.79	N/A	0–27
CEA (ng/mL)	1.16	N/A	0–5
ACTH (pmol/L)	6.38	N/A	0.7–7.0
Cortisol (nmol/L)	395.00	N/A	101.2–535.7
UFC (nmol/24 h)	818.06	N/A	138.2–1207.87
17-Hydroxyprogesterone (ng/mL)	6.45	N/A	Follicular phase: ≤1.05 Ovulatory phase: 0.13–1.46 Luteal phase: 0.27–2.41
DHAES (μg/dL)	116	N/A	94.8–513.7

ACTH = adrenocorticotropic hormone, AMH = anti-Mullerian hormone, CA125 = carbohydrate antigen 125, CA199 = carbohydrate antigen 199, CEA = carcinoembryonic antigen, DHEAS = dehydroxy epiandrosterone sulfate, HE4 = human epididymal protein 4, N/A = not available, UFC = urinary free cortisol.

**Figure 1.**
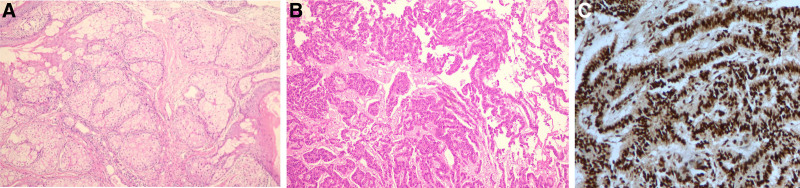
Ovarian teratoma (A, hematoxylin and eosin stain, ×200)-showing skin, sebaceous glands and cartilage. Sertoli Leydig cell tumor (B, hematoxylin and eosin stain, ×200)-showing tubular and adenoid structures. Immunohistochemical studies (C, inhibin-α immunostain, highlights the Sertoli Leydig cells, ×100).

**Figure 2.**
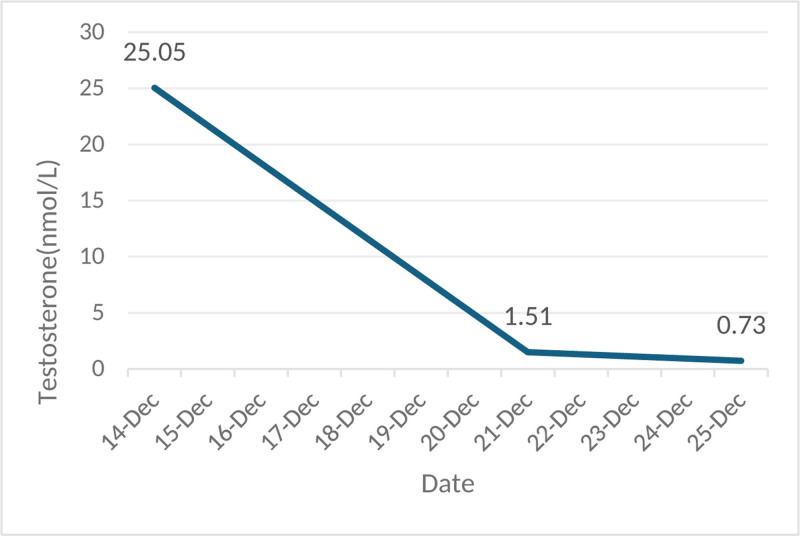
Line chart of testosterone level.

The patient underwent laparoscopic surgery to remove bilateral ovarian neoplasms on December 20, 2023. Among them, the left ovarian mass M1 and the right ovarian mass were mainly cystic, whereas the left ovarian mass M2 was mainly solid (4 × 4.5 × 4 cm^3^). The frozen response of the left ovarian mass M2 revealed that it was a sex cord-stromal tumor, and SLCT was considered. Postoperative pathological examination revealed that the left ovarian mass M2 was an SLCT with some teratoma components. It was moderately to highly differentiated. The left ovarian mass M1 and the right ovarian mass were MCTs. Immunohistochemical studies revealed that ovarian SLCTs expressed CK, Vim, inhibin-α, CD10, CD99 and MelanA to varying degrees (Fig. 1A–C). Testosterone decreased to 1.51 nmol/L on the 1st day after fertility-sparing bilateral ovarian tumor resection and to 0.73 nmol/L on the 5th day (Fig. 2).

## 3. Discussion

Mature teratomas of the ovary are common reproductive system tumors that are mostly asymptomatic and are usually discovered by chance during abdominal or pelvic surgery due to imaging examination, pregnancy, or other reasons.^[5]^ When the size of the tumor is too large, it can increase the pressure on the pelvis and cause acute abdominal pain, and teratomas with excessively large volumes can easily lead to ovarian pedicle torsion, which can even cause teratoma rupture, resulting in acute peritonitis that can develop into chronic granulomatous peritonitis in rare cases.^[6]^ When the expression of tumor markers such as CA199 increases, the malignant transformation of mature teratomas may occur.^[1,7]^ Surgical resection is an effective treatment for mature ovarian teratomas.^[1]^

In cases of ovarian SLCT, 40% to 60% of patients have amenorrhea, voice hoarseness, hirsutism, clitoromegaly and other masculinization phenomena.^[4]^ These characteristics are due to an increase in androgen production by tumor cells.^[8]^ Its diagnosis is based on changes in hormone levels, abdominal symptoms, and pathological examination results.^[9]^ The ability of Sertoli cells and Leydig cells to differentiate under a microscope is a necessary diagnostic factor.^[4]^

Here, we present a rare case of bilateral MCTs with left SLCT accompanied by elevated testosterone levels. The main manifestations of this patient were amenorrhea and an abdominal mass. The combination of clinical symptoms such as hirsutism, facial acne, amenorrhea, and laboratory test results suggested hyperandrogenism. In particular, laboratory tests revealed that plasma testosterone levels were elevated to at least 12.7 times the normal value. Imaging examination revealed a mass of undetermined nature on the left side with bilateral MCTs. On the basis of her clinical and examination results, a preliminary diagnosis of an androgen-secreting ovarian neoplasm was made. The androgens in women’s bodies are derived mainly from the adrenal glands and ovaries. When patients have hyperandrogenism, we need to distinguish whether the increase in androgen is related to the adrenal gland or ovaries. The main causes of hyperandrogenism caused by the adrenal glands include primary adrenal tumors, tumors that produce adrenocorticotropic hormones (such as Cushing’s disease), and congenital adrenal hyperplasia.^[10]^ 17-OHP, adrenocorticotropic hormone stimulation tests and dehydroxy epiandrosterone sulfate are laboratory indicators used to exclude adrenal hyperandrogenemia.^[4,11]^ The most common cause of hyperandrogenemia caused by ovarian factors is polycystic ovary syndrome (PCOS).^[10]^ Research has shown that elevated serum anti-Mullerian hormone levels can be used as a diagnostic marker of PCOS.^[12]^ However, the symptoms and signs of virilization are more likely to indicate that ovarian-derived tumors cause hyperandrogenemia. The incidence of hyperandrogenemia caused by sex-related stromal tumors is 91%.^[13]^ The 17-OHP level of this patient was mildly elevated, and the cortisol and dehydroxy epiandrosterone sulfate levels were within the normal range; no adrenal hyperplasia was observed on contrast-enhanced magnetic resonance. According to Zeng and colleagues, patients with ovarian or adrenal tumors can have elevated 17-OHP levels.^[14]^ Therefore, congenital adrenal hyperplasia was not considered. At present, there are no examination results to support the diagnosis of adrenal tumors and PCOS, and CA125, CA199 and HE4 levels are within the normal range. However, imaging examination revealed an ovarian tumor of an undetermined nature, and her hyperandrogenemia may be related to this. The postoperative pathological report confirmed the diagnosis. Therefore, the possibility of SLCT should be highly suspected in young female patients with masculine features, pelvic masses, and elevated plasma testosterone levels after ruling out adrenal hyperplasia. The patient’s plasma testosterone level returned to normal on the 1st day after bilateral ovarian tumor removal. Once an ovarian SLCT is diagnosed, it should be surgically removed immediately. The combination of chemotherapy or radiotherapy was selected in this case according to the immunohistochemical results.

## Author contributions

**Data curation:** Jie Luo.

**Formal analysis:** Shimei Li.

**Supervision:** Ping Sun.

**Validation:** Yulan Song.

**Writing – original draft:** Yingtong Zhang.

**Writing – review & editing:** Yulan Song, Yingtong Zhang.
